# Challenges in developing a split drive targeting *dsx* for the genetic control of the invasive malaria vector *Anopheles stephensi*

**DOI:** 10.1186/s13071-025-06688-0

**Published:** 2025-02-07

**Authors:** Mireia Larrosa-Godall, Joshua X. D. Ang, Philip T. Leftwich, Estela Gonzalez, Lewis Shackleford, Katherine Nevard, Rob Noad, Michelle A. E. Anderson, Luke Alphey

**Affiliations:** 1 Arthropod Genetics, The Pirbright Institute, Pirbright, GU24 0NF UK; 2 Department of Biology, University of York, Wentworth Way, York, YO10 5DD UK; 3 York Biomedical Research Institute, University of York, Heslington, YO10 5DD UK; 4 Current Address: Animal and Plant Health Agency, Woodham Lane, Addlestone, Surrey KT15 3NB UK; 5 School of Biological Sciences, University of East Anglia, Norfolk, Norwich, NRA 7TJ UK; 6 Current Address: Pathobiology and Population Sciences, The Royal Veterinary College, Hawkshead Lane, Brookmans Park, Hatfield, AL9 7TA UK

**Keywords:** CRISPR/Cas9, Gene drive, Doublesex, Malaria, *Anopheles stephensi*

## Abstract

**Background:**

*Anopheles stephensi* is a competent malaria vector mainly present in southern Asia and the Arabian Peninsula. Since 2012, it has invaded several countries of eastern Africa, creating an emerging risk of urban transmission. Urgent efforts are required to develop novel and more efficient strategies for targeted vector control. CRISPR/Cas9-based homing gene drives have been proposed as attractive alternative strategies. Gene drives have the potential to spread a desired trait through a population at higher rates than via normal Mendelian inheritance, even in the presence of a fitness cost. Several target genes have been suggested and tested in different mosquito vector species such as *Anopheles gambiae* and *Aedes aegypti*. Several promising suppression drives have been developed in *An. gambiae* that target the sex determination gene *doublesex* (*dsx*).

**Methods:**

In this study, a geographically confineable gene drive system targeting *dsx* was developed (*dsx*^*gRNA*^). Here, a transgenic line which expresses Cas9 under the control of the endogenous *zpg* promoter was generated. Separately a transgenic line which expresses a gRNA targeting the female specific exon of *dsx* was inserted into that same target site. The reproductive fitness of males and females heterozygous and homozygous for this element was determined. A series of experimental crosses was performed to combine the two elements and assess the homing rate of the *dsx* element in a split drive system.

**Results:**

The drive was able to home in a super-Mendelian rate comparable to those obtained by an autonomous drive in this species. Although inheritance rates as high as 99.8% were observed, potentially providing very potent gene drive, dominant effects on male and female fertility were observed, which would be sufficient to hinder spread of such a drive. Molecular analysis indicated that the gRNA expressing insertion disrupted normal splicing of *dsx*.

**Conclusions:**

These results should be considered when proposing the viability of *dsx* as a target gene for a population suppression gene drives in *Anopheles stephensi*. Although high homing rates were observed, the fitness defects found in both males and females carrying the transgene would likely prohibit this drive from functioning in the field.

**Graphical Abstract:**

**Figure:**
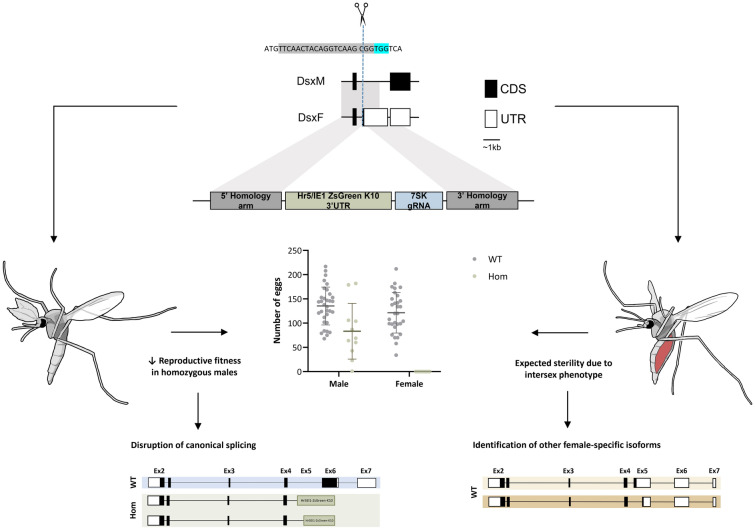

**Supplementary Information:**

The online version contains supplementary material available at 10.1186/s13071-025-06688-0.

## Background

*Anopheles stephensi* is a highly competent urban malaria vector with high susceptibility to both *Plasmodium falciparum* and *Plasmodium vivax* [1]. Until 2012, it was known for its role in malaria transmission in countries including India, Iran, Iraq, Pakistan and Afghanistan where it has been the main vector [2]. Since then, it has invaded the African continent where it continues to spread rapidly [1]. The greater adaptability of *An. stephensi* to urban environments compared to the more rural main malaria vectors in Africa (*Anopheles gambiae* and *Anopheles funestus*) represents a problem for malaria transmission in urban areas where malaria transmission has previously been low or non-existent. *Anopheles stephensi* is thought to have contributed to a resurgence of malaria cases in Djibouti City [3–5] and at least one outbreak in Ethiopia [6]. Given these observations, if *An. stephensi* continues to spread unchecked, it could undermine the advances made towards malaria elimination on the continent, increasing the population at risk by 126 million [7]. Although measures targeting the mosquito vectors have proven to be the most effective for reducing malaria, the current measures are insufficient to achieve eradication. Therefore, successful interventions towards disease eradication require novel and more efficient technologies such as CRISPR/Cas9-based homing gene drives.

Homing gene drives are selfish genetic elements that are able to drastically bias their own inheritance [8, 9]. Thus, they can rapidly spread within a population even at a low introduction frequency and while carrying a fitness cost, making them a promising tool to reduce the burden of vector-borne diseases. Because of these properties, homing gene drives are potentially a self-sustaining strategy for vector control which relies on mating, making them species-specific, environmentally friendly and very cost-efficient. Self-sustaining homing-based gene drives for population suppression and population replacement have been successfully generated in *Anopheles* mosquitoes [10–14], potentially providing novel and efficient tools for malaria control. However, the fact that such autonomous homing gene drives can spread to any population that shares genetic flow with the target population, and therefore potentially the entire species, comes with environmental, regulatory and ethical concerns. One approach to limit the spread of a gene drive into nearby populations is to engineer a system that is only able to drive and successfully alter a population and to make its drive property dependent on another, non-driving component. The non-driving component then acts as a ‘licensing factor’ or ‘tether’ for the drive, which will only spread if the licensing factor is present at significant frequency, i.e. where it has been deliberately released. More precisely, the driving component(s) will increase in frequency if drive is frequent enough to outweigh the fitness cost of the drive component(s). Furthermore, such drives are self-exhausting, since the non-driving component will gradually decrease in frequency, assuming at least some fitness cost, unless periodically supplemented by additional releases. Thus, migration and/or accidental release of gene drive mosquitoes into a nearby population should not have a significant impact. This could be achieved by separating the guide RNA (gRNA) and Cas9 components of the CRISPR/Cas9 system (split drive) inserted in different loci, one of which does not home [9, 15]. Homing then occurs only in individuals heterozygous for the homing element, which also carry at least one copy of the non-homing ‘licensing factor’.

*doublesex* (*dsx*) is a key gene in the sex determination cascade responsible for the differentiation of sexually dimorphic morphological traits in both males and females. First identified in *Drosophila melanogaster, dsx* is alternatively spliced to produce male (*dsxM*) and female (*dsxF*) transcripts [16]. It has been proposed as a target gene for population suppression gene drives since disruption of the female-specific isoform of *dsx* (*dsxF*) in several insect species results in a sterile, intersex phenotype [17–22]. In *An. gambiae*, disruption of dsxF through the insertion of a GFP-expressing cassette into the female-specific exon of *dsx* also caused a sterile intersex phenotype in homozygous females without altering the development or fertility of heterozygous females or males [10]. The addition of Cas9 and gRNA expression cassettes to the *dsxF* insertion resulted in production of very high inheritance rates of the transgene, allowing the drive to suppress a caged population when released at 12.5% allele frequency [10]. Following cutting by CRISPR/Cas9, the double-stranded DNA break can be repaired by homology-dependent repair, leading to homing. However, end-joining, an alternative repair pathway, can lead to cut-resistant mutations at the target site. These can arise at much higher rates than normal spontaneous mutation and may inhibit the spread of a homing-based drive, especially if they maintain function of the target sequence [8, 23–26]. However, no functional mutations were detected in any of the cage trials performed with the dsxF^CRISPRh^ mosquito line [10, 27], indicating that *dsx* is a highly conserved sequence where cut-resistant mutations have an associated fitness cost.

Two challenges of gene drive research are target site resistance and modulation of their invasive potential. In this study, we aim to confront these challenges by developing a geographically confineable gene drive system that allows a local and temporal control of an *An. stephensi* mosquito population by targeting *dsx*, a highly conserved gene which has been demonstrated to function as a population suppression gene drive in *An. gambiae*. To develop a split-drive capable of population suppression, we developed a construct similar to the *dsxF* construct developed by Kyrou et al. [10] with the addition of a gRNA targeting the 5’ intron-exon boundary of the *dsx* female-specific exon. We examined the resulting phenotype and the ability of the construct to be driven to high inheritance rates in a split drive system.

## Methods

### Plasmid design

The AGG2073 plasmid (*dsx*^*gRNA*^) was designed to express a gRNA under the control of the *As*7SK promoter (ASTE015331) and a fluorescent marker (Hr5/IE1-ZsGreen-K10) flanked by 1-kb homology arms. The 7SK RNA is an abundant small RNA which acts as a regulator of transcription by interacting with elongation factors in the nucleus. Unlike U6 RNA, which has multiple copies across the genome, 7SK is a single copy non-coding RNA gene, expressed in all transcriptionally active cells. The gRNA was specifically chosen to target the splicing acceptor of the female-specific exon of the *Asdsx* gene (ASTE008815) as previously reported by Kyrou et al. [10]. Upstream and downstream sequences of the target site were used to establish the sequence of both homology arms. The full plasmid sequence can be retrieved from NCBI (PQ306471).

The AGG1760 plasmid was designed to express Cas9 under the control of the endogenous *vasa* promoter and a 3xP3-mCherry-SV40 fluorescence cassette contained within *piggyBac* transposable element flanks (PQ306470). This plasmid was utilised as a helper plasmid when injecting the AGG2288 plasmid into *An. stephensi* embryos.

The Cas9 was inserted at the N-terminus of Zpg (ASTE011088) to utilise the endogenous *zpg* promoter, which is known to direct the expression of the Cas9 protein to the pole cells of the embryo in development in *An. gambiae* [28]. We inserted an insect codon optimised Cas9 [29] after the first five amino acids of Zpg. The 3xP3-mCherry-K10 marker cassette is contained within a synthetic intron [30] inserted into the NLS at the 3’ end of Cas9 coding sequence. The ORF of Cas9 is followed by a ubiquitin monomer which should result in the cleavage of the Cas9 from Zpg, leaving the ubiquitin on the C’ of Cas9. Also included outside of the homology arms is a cassette to express the gRNA CAGAATGTTCTGCAACGGTCTGG (PAM underlined) using the *As*7SK promoter for HDR insertion. The resulting plasmid was AGG2288 (*zpg*^*5’Cas9*^). The full plasmid sequence is available through the NCBI accession no. PQ306472.

### Mosquito rearing

*Anopheles stephensi* mosquitoes of the SDA-500 (WT) strain as well as mosquitoes expressing the *dsx*^*gRNA*^ and *zpg*^*5’Cas9*^ transgenes were reared in environmentally controlled rooms set at standard conditions (28 ± 1 oC, 70–85% relative humidity, and a 14:10 day-night cycle). First-instar larvae were reared in pools of 200 per tray containing 500 ml of RO water and fed with resuspended dry food (Seramicron, Sera) or fish food pellets (ExtraSelect, Su-Bridge Pet Supplies, Ltd.) at later larval stages. Adult mosquitoes were provided with 10% sucrose ad libitum. Adult females were additionally fed once a week on defibrinated horse blood (TCS Bioscience) supplied through Hemotek membrane feeders (Hemotek Ltd.) and a double layer of Parafilm (Bemis) membrane.

### Generation of transgenic mosquito lines

*Anopheles stephensi* mosquitoes of the SDA-500 strain (WT) were reared in a climate-controlled room with a reversed day-night cycle. Females were blood-fed with horse defibrinated blood (TCS Bioscience), and freshly laid eggs were aligned for microinjections as previously described [31]. To generate *dsx*^*gRNA*^ mosquitoes, eggs were injected with an injection mix containing 1X injection buffer [32], 300 ng/ul Cas9 (PNABio), 50 ng/ul in vitro transcribed gRNA [33] and 800 ng/ul AGG2073 donor plasmid. The injection mix for the *zpg*^*5’Cas9*^ contained 1X injection buffer [32], 300 ng/ul of a helper plasmid expressing Cas9 (AGG1760) and 300 ng/ul of the AGG2288 donor plasmid. Surviving G_0_s were maintained at standard conditions until adulthood when females and males were separately crossed in pools of 20 G_0_ mosquitoes to SDA-500 in 1:2 and 5:1 ratios, respectively. Females were blood-fed with defibrinated horse blood (TCS Bioscience) to obtain G_1_ larvae, which were screened for the fluorescent marker using a Leica MZ165C fluorescence microscope. Fluorescent G_1_ larvae were individually crossed to WT to generate G_2_ progeny. Details of injection survivors and transformation rate are presented in Table S1. Transgene insertion for the *dsx*^*gRNA*^ was confirmed by PCR amplification of gDNA using LA4340-LA6560 and LA5160-LA4348 primer pairs, whereas the LA4756-LA6629 and LA4902-LA4760 primer pairs were used for the *zpg*^*5’Cas9*^ transgene. The PCR was followed by Sanger sequencing. Complete primer sequences can be found in Table S12.

### External sex characterization

Female and male homozygotes, heterozygotes and WT siblings of the *dsx*^*gRNA*^ line were collected as pupae and adults, anaesthetized with CO_2_ and placed on ice under a Leica MZ165C microscope. For better visualisation of the phenotype, the legs of adult mosquitoes were removed. Images of the different phenotypes were captured using a Leica DFC7000T camera. Whole body images were taken using 22.5 ms exposure, gain 1 and 0.7 × magnification. The magnification was increased for close-up images of the external genitalia to 3.8×.

### Fecundity and fertility assays

To determine the reproductive phenotype of mosquitoes carrying the *dsx*^*gRNA*^ allele, *dsx*^*gRNA*^ heterozygous females and males were sibling crossed in a 1:1 ratio. The progeny were sorted using a Biosorter (Union Biometrica) to separate homozygotes, heterozygotes and WT according to fluorescence intensity. Larvae were reared under the same conditions until adulthood. Mosquitoes were anaesthetized with CO_2_ to facilitate the extraction of the posterior left leg, which was used to characterize their sex and genotype by PCR using the Phire Animal Tissue Direct PCR kit (ThermoFisher Scientific) and primers LA7518 and LA7520. PCR-confirmed homozygous, heterozygous and WT males and females were outcrossed to WT mosquitoes of their opposite sex. Their fecundity and fertility were assessed using EAgaL plates [34].

### Assessing the CRISPR/Cas9-induced inheritance bias

*dsx*^*gRNA*^ heterozygous males and females were crossed to *zpg*^*5’Cas9*^ heterozygous females and males respectively (F_0_). Their offspring (F_1_) were screened under a Leica MZ165C fluorescence microscope, and trans-heterozygous males and females for the *dsx*^*gRNA*^ and *zpg*^*5’Cas9*^ alleles were retrieved and outcrossed to WT mosquitoes of the opposite sex in a 1:1 ratio. After a blood meal, females were placed in individual cups where they were allowed to lay eggs. The number of embryos laid per female was counted. Hatched larvae were screened for the presence of the ZsGreen fluorescent marker at L3–L4 stage under a Leica MZ165C fluorescence microscope to determine the inheritance rate of the *dsx*^*gRNA*^ transgene.

### Statistical analysis

Fecundity was analysed with a generalised linear mixed model with a zero-inflated Poisson error distribution (model selection was determined through AIC scores and checks of simulated residuals). Fixed effects included the line and the sex of the Cas9-bearing parent with a random effect of individual parent to account for batch effects and overdispersion. Fertility and homing rates were both analysed with a generalised line mixed model with a binomial (logit) error distribution; fixed effects included line and the sex of the Cas9 bearing parent with a random effect of individual parent to account for batch effects and overdispersion. Homing models also included a fixed effect of the sex of the Cas9-bearing grandparent. All models were constructed in R version 4.4.1 using the package glmmTMB, emmeans for mean estimates and 95% confidence intervals, and ggplot2 for data visualisation. Raw data and analysis scripts are available at https://github.com/Philip-Leftwich/split-drive-dsx-Anopheles-stephensi.

### Characterization of the *dsx* canonical splicing in *An. stephensi*

RNA extracted with the NucleoSpin RNA kit (Macherey-Nagel) from pools of 10 *dsx*^*gRNA*^ homozygous and WT male and female adults was used as a template to synthesise 5’ and 3’ RACE ready cDNA. The SMARTer RACE 5’/3’ Kit (Takara Bio) was used for the generation of RACE ready cDNA and for the RACE PCR. Complete primer sequences are listed in Table S12. The obtained amplicons were cloned into a pJET vector using the CloneJET PCR cloning kit (ThermoFisher Scientific), Sanger sequenced and aligned to the genome assembly of *An. stephensi* SDA-500 strain. Raw sequences are available for download from the research data repository of the University of York at https://doi.org/10.15124/77dd2bf5-06a6-4878-8f17-3423c1a98480 [35].

## Results

### Building a population suppression split drive targeting *AsdsxF*

**Fig. 1:**
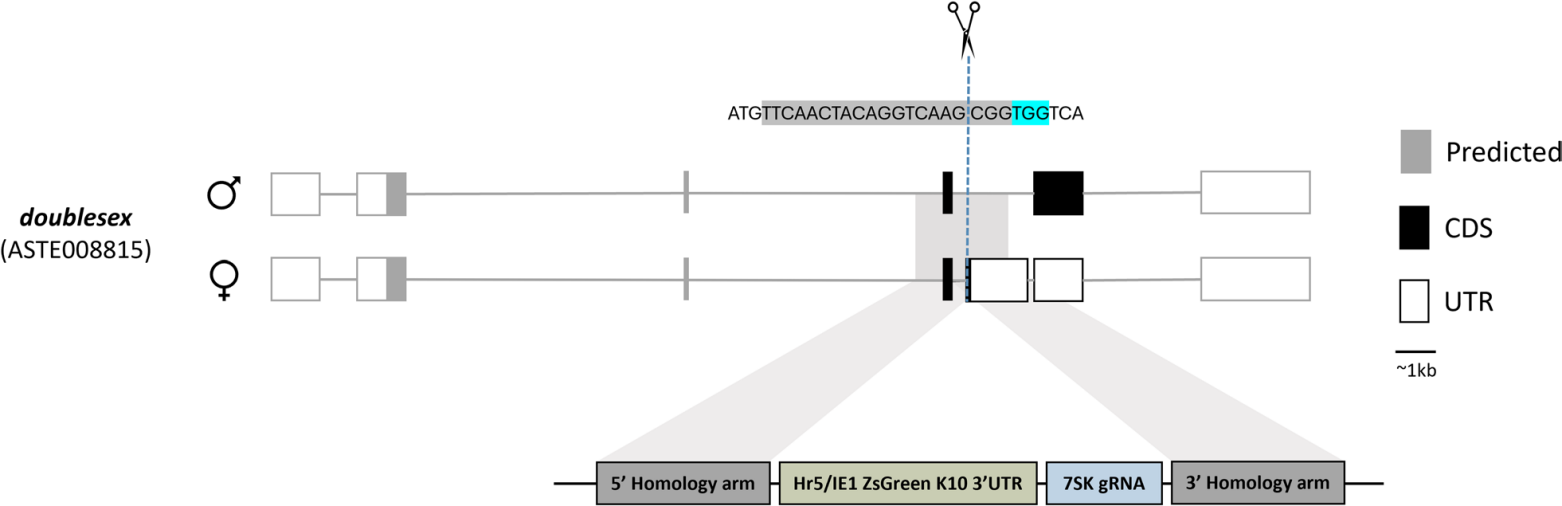
Development of a split drive targeting *dsxF*. Schematic representation of the male- and female-specific *dsx* transcripts in *Anopheles stephensi* including the gRNA sequence and the HDR knock-in construct with its corresponding insertion site locus. The black region of the *Asdsx* transcript was retrieved from Vectorbase (ASTE008815), whereas the remaining sequence (grey) was predicted from homology to *Anopheles gambiae* (AGAP004050). The expressed gRNA was designed to target the intron 4-exon 5 boundary. The cut site of the gRNA is indicated by scissors and the protospacer-adjacent motif (PAM) is highlighted in blue. Exons are drawn approximately to scale, whereas introns are not.

Disruption of the female-specific isoform of *Asdsx* was achieved via insertion of marker and gRNA expression cassettes through homology-directed repair (HDR) (Fig. 1). The HDR donor plasmid (AGG2073) consisted of a gRNA expressed by the endogenous 7SK promoter and the ZsGreen fluorescent marker under the control of the of Hr5/IE1 (*homologous region 5* enhancer fused with the *immediately early 1* gene) promoter and the *K10* (*fs*(1) K10) terminator, flanked by 2-kb homology arms aligning to the immediate upstream and downstream regions of the gRNA cut site. Seven hundred twenty-two *An. stephensi* embryos were injected with the donor plasmid (AGG2073), Cas9 protein (PNABio) and an in vitro transcribed gRNA. Two of the 19 surviving G_0_ females presented an intersex phenotype, showing evidence of mosaicism. All G_0_ adults were outcrossed to WT mosquitoes and their progeny were screened for the presence of the fluorescent marker; 159 G_1_ larvae were identified with the ZsGreen transformation marker (Table S1), which were used to generate different isolines. HDR-mediated integration of each isoline was confirmed by PCR amplification using primers inside and outside of the transgene (Fig. S1). A single sequence-confirmed isoline was selected to be maintained as the *dsx*^*gRNA*^ transgenic line.

**Fig. 2:**
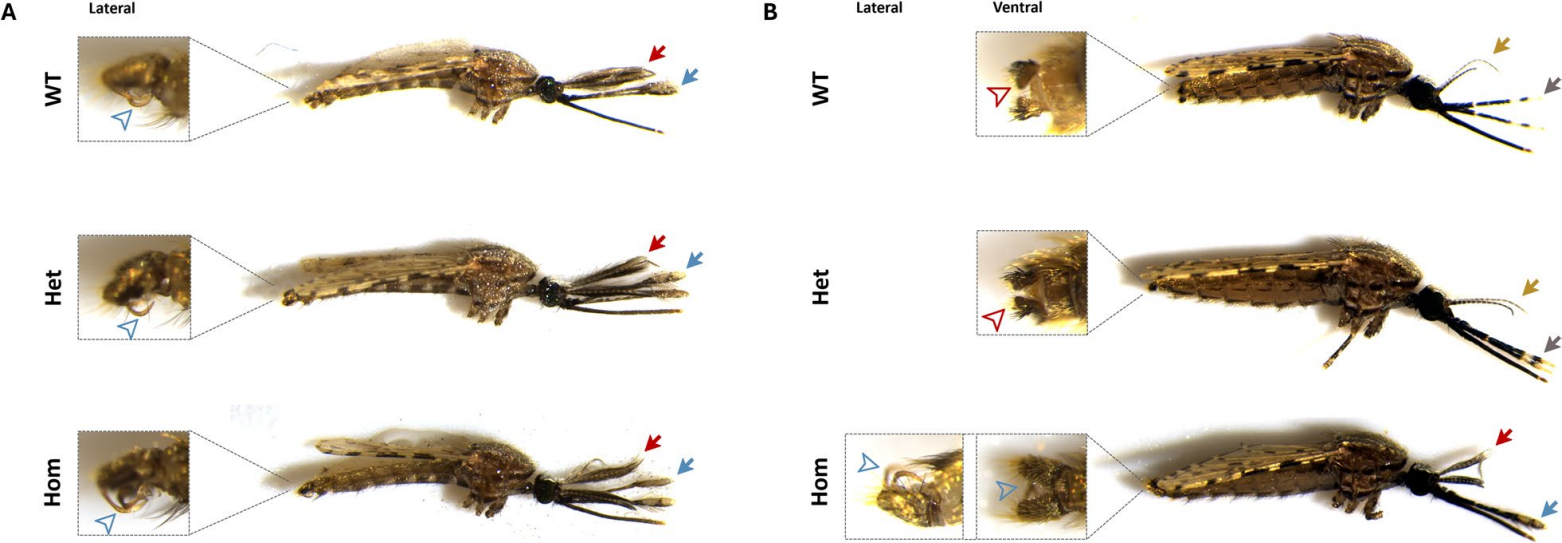
Disruption of the female-specific isoform of the *Asdsx* gene by homology-directed repair results in a female-specific intersex phenotype. Morphological appearance of *dsx*^*gRNA*^ homozygotes, heterozygotes and WT adult siblings. **A** Image of adult males with specific emphasis on the external genitalia (blue arrowhead) from a lateral view. The red arrow indicates the antenna and the blue arrow the palps. **B** Image of adult females. Heterozygous and WT females presented female-like features: pilose antenna (yellow arrow), narrow palps (grey arrow) and cerci (red arrowhead). Homozygous females presented both male and female morphological characteristics: plumose antennae (red arrow), dorsally rotated claspers (blue arrowhead) and their palps (blue arrow) resembled those of males. External genitalia were imaged from a ventral view for all the genotypes and lateral view for homozygous females to appreciate the direction of the claspers.

To assess whether the generated *dsx*^*gRNA*^ line showed the same phenotype as previously observed in *An. gambiae* [10], *dsx*^*gRNA*^ heterozygotes were intercrossed. This cross generated WT, heterozygous and homozygous mosquitoes for the *dsx*^*gRNA*^ allele at the expected Mendelian ratio of 1:2:1 (Table S2, Table S3 and Fig. S2). Heterozygous and WT individuals developed into females and males in a sex ratio close to 1:1 as expected (Table S4). Although all homozygotes were identified as males as pupae (Fig. S3), half of them developed into normal-appearing males (Fig. 2A) and the other half developed into adults with both male- and female-like morphological traits (intersex phenotype) (Fig. 2B). The sex chromosome composition of the intersex individuals was characterised by PCR amplification of GUY1, a gene located on the male-specific Y chromosome [36]. PCR amplification of GUY1 showed that all the adult mosquitoes that presented an intersex phenotype were genetically females. No significant differences were observed in the transgenic or sex ratio (Table S4 and Fig. S2), suggesting that the transgene had no significant effect on larva to adult viability.

Close examination of external sexually dimorphic features in homozygous females showed that all presented an intersex phenotype, with several abnormalities. They presented plumose antenna with longer flagellomeres instead of the pilose female-specific antenna, palps which widened at the most anterior end and claspers instead of the female-specific short cerci. However, the claspers failed to rotate to the ventral position (Fig. 2B). All these traits are categorised as male-specific characteristics and were also reported in homozygous *An. gambiae* females with disrupted *dsxF* [10]. These morphological abnormalities were absent from heterozygous females (Fig. 2B), suggesting that *dsx* might be a haplosufficient gene in *An. stephensi*, as previously reported for *An. gambiae* [10].

Even though only homozygous females appeared to present morphological differences compared to their WT siblings, we examined the fertility and fecundity of the male and female mosquitoes expressing the *dsx*^*gRNA*^ transgene. Heterozygous *dsx*^*gRNA*^ were intercrossed to obtain homozygotes, heterozygotes and WT males and females, which were then outcrossed to SDA-500 mosquitoes of the opposite sex in a 1: 1 ratio. Mated females were offered a blood meal, and the number of eggs laid and the number of hatched larvae from each female progenitor was scored.

**Fig. 3:**
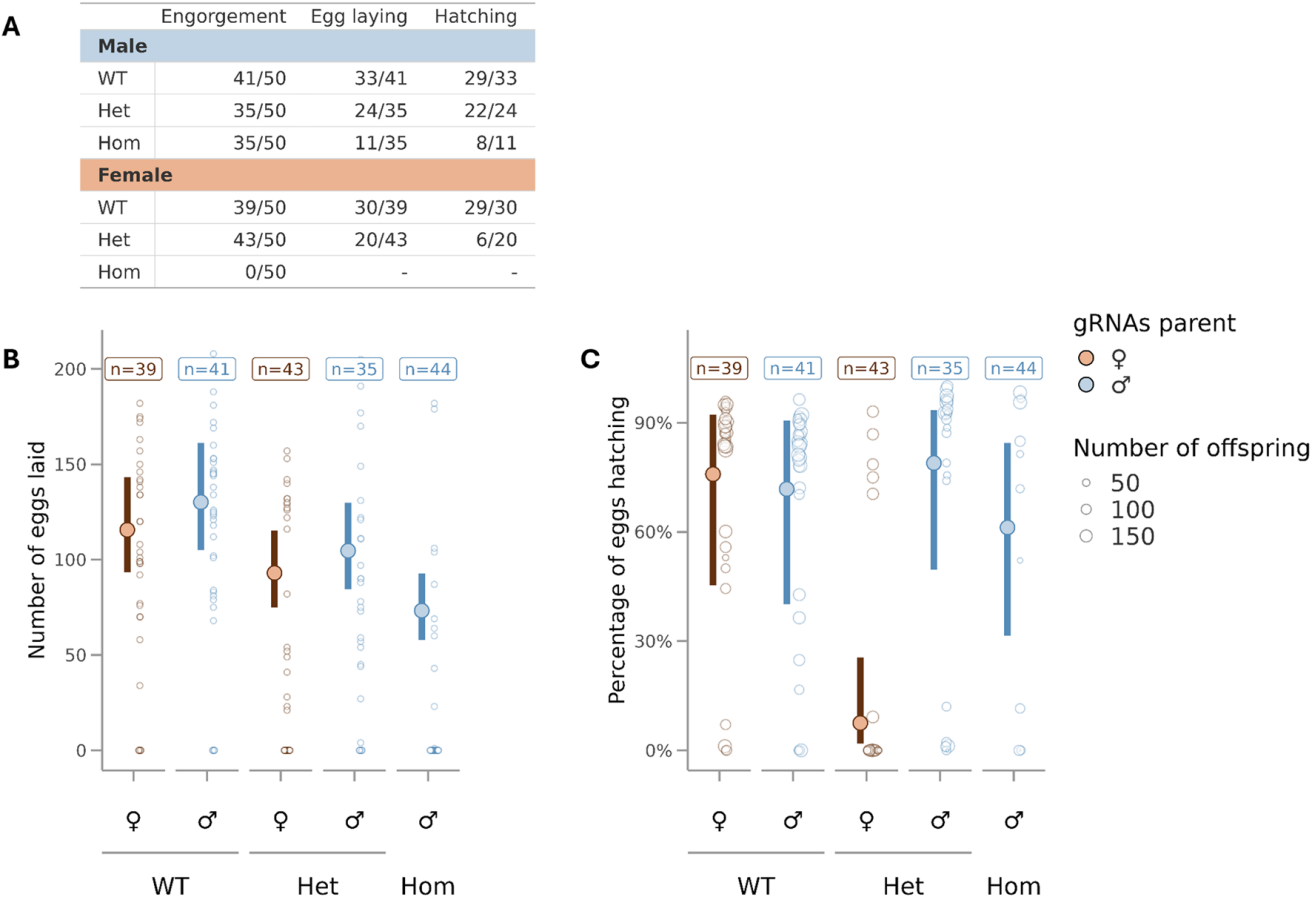
Analysis of the reproductive fitness of mosquitoes carrying the dsx^*gRNA*^ allele. **A** Itemization of all stages between blood-feeding and hatching rate that contribute to reproductive fitness. In each stage, females that were unable to contribute were excluded from the next. **B** Number of eggs that were laid per female. **C** Hatching rate of the eggs laid by each female (number of hatched larvae was divided by the number of eggs and multiplied by 100). *WT* wild type, *Het* heterozygote, *Hom* homozygote. *n* = the number of individuals whose progeny were scored.

We analysed the probability of laying no eggs at all and the mean number of eggs produced per engorged female. Although most heterozygous and WT females as well as the SDA-500 females crossed to homozygous, heterozygous and WT males were able to blood-feed; *dsx*^*gRNA*^ homozygous females were not able to ingest and retain a blood meal (Fig. 3A), resembling the phenotype observed in *An. gambiae* [10]. Non-engorged females were excluded from the fecundity and fertility assays because they were unable to lay eggs. From all the engorged females scored, SDA-500 crosses to WT had an estimated probability of not laying eggs of 20.9% [95% CI 13.3–31.3] but this increased to 42.6% [32%–54%] (*z* = 2.88, *p* = 0.004) and 75% [60%–85%] (*z* = 5.6, *p* < 0.001) for SDA-500 crosses to heterozygotes and homozygotes respectively. We found no evidence for a difference in fecundity based on the direction of the mating cross (*z* = 1.737, *p* = 0.08) and no evidence for a genotype specific effect on the direction of the mating cross (*z* = 0.975, *p* = 0.32). There was an effect of genotype on the mean number of eggs laid, and while the number of eggs laid from crosses with heterozygotes (98.7 [83–117]) was lower than wild type (121.4 [102–144]), this was not statistically significant (z = −1.44, *p* = 0.15). However, SDA-500 females that were crossed to homozygous males laid significantly fewer eggs (73.3 [58—92.6]) (z = −3.39, *p* < 0.001) (Fig. 3B and Table S5).

Heterozygous *dsx*^*gRNA*^ females laid eggs that had a significantly lower hatch rate (7.5% [1.8–255] (z = −2.91, *p* = 0.004)) than wild-type crosses (73.9% [52.4–87.9]); no other significant changes in hatching rates were observed for the other crosses (Fig. 3C and Table S6).

### Compatible pairing of a Cas9-expressing line with the generated dsx^*gRNA*^ line can induce high levels of biased inheritance

**Fig. 4:**
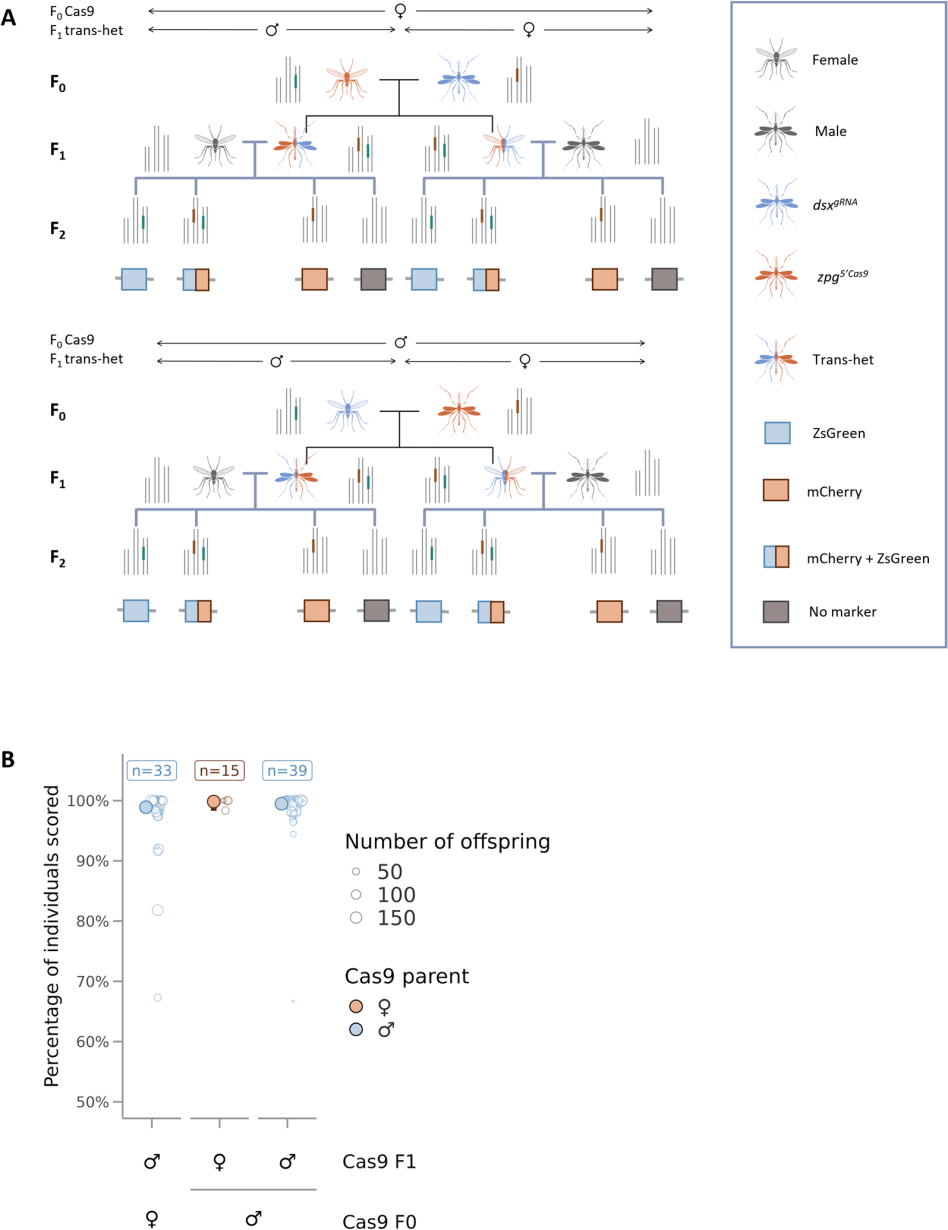
Near 100% inheritance bias of *dsx*^*gRNA*^ was observed in the presence of *zpg*^*5’Cas9*^. **A** Scheme of the crosses performed for the assay representing the four different crossing directions. **B** Percentage of F_2_ larvae that inherited the *dsx*^*gRNA*^ element. Open circles represent the inheritance rates from a single female and their size is proportionate to the number of offspring. The progeny of trans-heterozygous females is represented in orange and the offspring of trans-heterozygous males in blue. Filled points and error bars represent the mean and the 95% CI. *n* = the number of individuals whose progeny were scored.

To determine whether inheritance rates comparable to those obtained for the *dsxF*^*CRISPRh*^ line in *An. gambiae* [10] could be achieved in a split drive system, *dsx*^*gRNA*^ heterozygotes were crossed to heterozygotes of a transgenic line expressing Cas9 under the control of the endogenous *zpg* promoter (*zpg*^*5’Cas9*^). This cross produced WT, *dsx*^*gRNA*^ heterozygotes, *zpg*^*5’Cas9*^ heterozygotes and trans-heterozygotes for the *dsx*^*gRNA*^ and *zpg*^*5’Cas9*^ alleles at the expected Mendelian ratio of 1:1:1:1 (Table S7), suggesting that expression of both Cas9 and gRNA in the same individual did not affect their survival. However, F_1_ trans-heterozygous females which obtained the Cas9 transgene from their female parent presented degrees of intersex phenotypes as pupae. Therefore, pupae with female-like features were selected for the assay to try to generate F_2_ offspring. No morphological differences were observed in F_1_ trans-heterozygous female pupae which received the Cas9 from the paternal progenitor. Male and female trans-heterozygotes were outcrossed to SDA-500 mosquitoes of the opposite sex and their progeny were screened for fluorescence to determine drive efficiency (Fig. 4A).

The fertility of the F_1_ trans-heterozygous males and females was also assessed to determine potential fitness effects due to parental deposition of the nuclease into the embryos and/or the expression of the nuclease in somatic cells. The number of engorged females, the number of dead females after blood-feeding, the number of females that laid eggs and the number of females whose eggs hatched were scored. A significant reduction in the fertility of female trans-heterozygotes was observed in all crosses. However, a greater negative impact on females’ fertility was observed when the Cas9 was inherited maternally (*z* = −5.433, *p* < 0.001) (Fig. S4). Presumably Cas9 deposited in the embryo resulted in some somatic cleavage of *dsxF*, causing most females to be unable to engorge (Table S8) and those who managed to blood-feed to be unable to lay any eggs (Table S8 and Fig. S4A). A similar but minor effect was observed in trans-heterozygous females which received the nuclease from the male progenitor (*z* = −2.1, *p* = 0.004) (Table S8 and Fig. S4), suggesting some somatic expression of Cas9. No apparent changes were observed in the reproductive phenotype of males that inherited the Cas9 either from the maternal or paternal parent.

The F_2_ offspring of trans-heterozygous females which inherited the nuclease paternally and trans-heterozygous males (with both maternal and paternal inheritance of Cas9) were screened for the presence of the *dsx*^*gRNA*^ transgene (Fig. 4B) and the *zpg*^5’Cas9^ transgene (Fig. S5). We observed 98.9% ([95% CI] = [97.9–99.4%]) of the offspring of trans-heterozygous males (maternally inherited Cas9) inherited the *dsx*^*gRNA*^ allele (Fig. 4B). Moreover, the drive was inherited by 99.5% ([95% CI] = [99–99.7%]) of the progeny of male trans-heterozygotes and 99.8% ([95% CI] = [98.3–100%]) of the progeny of female trans-heterozygotes that paternally inherited the Cas9 (Fig. 4B). These inheritance rates were significantly higher than the predicted Mendelian inheritance rates of 50% (z = 5.2, *p* < 0.001, *n* = 75).

### Disruption of *dsxM* and *dsxF* canonical splicing in the presence of the transgene might be associated with reduced fertility

We hypothesized that the reduced fertility of heterozygous males and females was due to the transgene so we performed rapid amplification of cDNA ends (RACE) to analyse *dsx* splicing. Annotation of the *Asdsx* gene in Vectorbase (ASTE008815) only consists of what we determined to be exons 4–6 by similarity to *Agdsx*. Therefore, first we performed 5’RACE to verify the 5’ end of the *dsx* transcript. We found an additional upstream non-coding exon in both males and females, which we refer to as Ex0. This exon was present as three different sequences (Ex0a, Ex0b or Ex0c). Moreover, three different isoforms of Ex1 were observed: the canonical isoform, an isoform which spliced from Ex1a to Ex1b and a third which only contained Ex1b (Fig. S6). No major differences were observed in the CDS except for Ex2, which had two different isoforms: one that splices from Ex2a to Ex2b and another one that reads through the intron (Fig. S6) similar to *Agdsx* [37]. These isoforms were observed in both males and females, suggesting that male and female isoforms of *Asdsx* have a common N-terminus. Alignment of the amino acid sequences of Ex2 showed that the small intron was in frame and codes for an additional 24 amino acids.

**Fig. 5:**
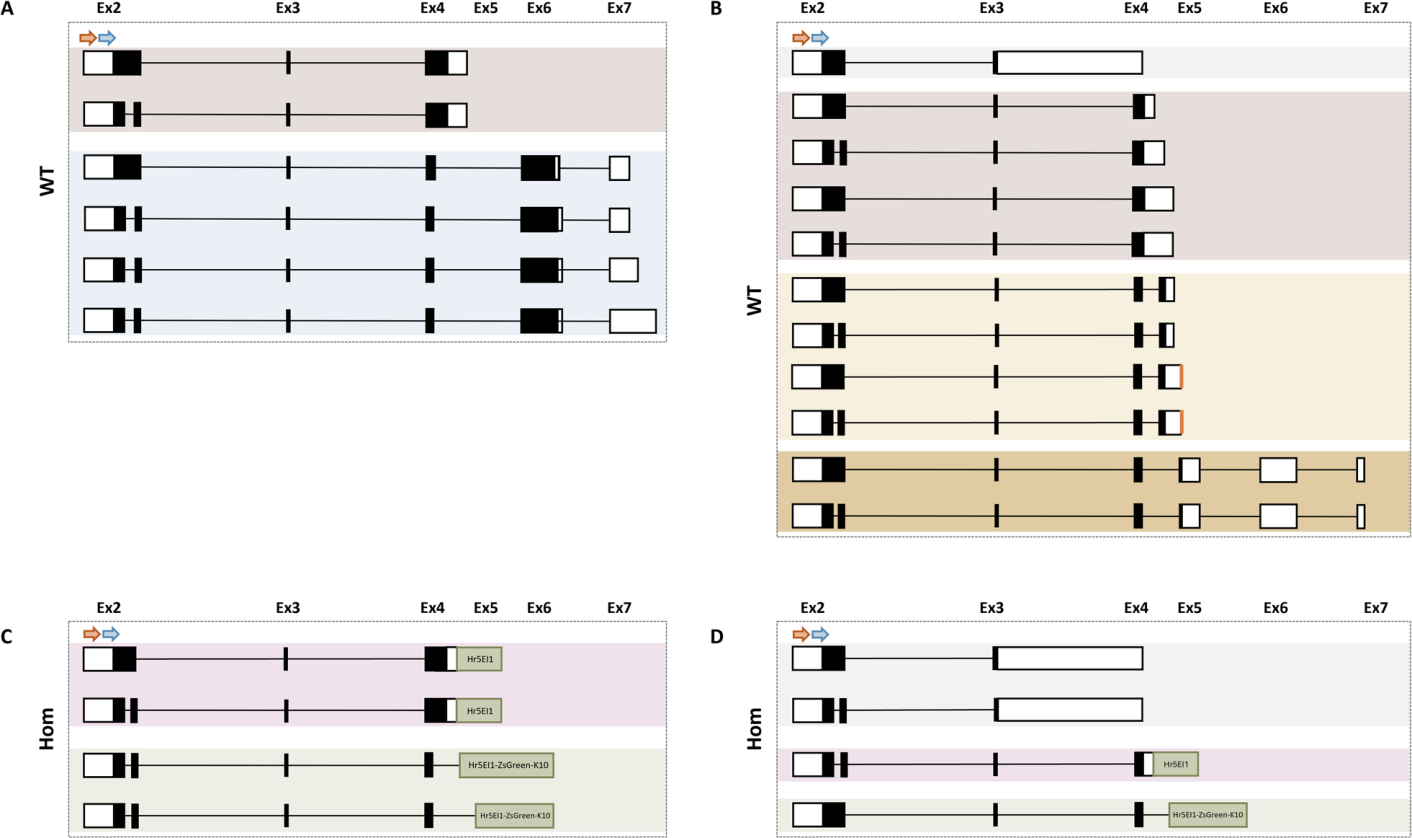
The *dsxM and dsxF* canonical splicing appeared to be disrupted in *dsx*^*gRNA*^ homozygous mosquitoes. **A** Isoforms identified for *dsxM* by RACE performed on the 3’ end of the cDNA transcript of homozygous *dsx*^*gRNA*^ and WT male mosquitoes. **B** Isoforms found for *dsxF* by RACE on the 3’ end of the cDNA transcript of WT and homozygous *dsx*^*gRNA*^ female mosquitoes. Only sequences that contained a poly A tail were included in the analysis. Coding sequences are represented in black, untranslated regions in white and the *dsx*^*gRNA*^ cassette in green. The orange arrow indicates the location of the GSP (LA8222) and the blue arrow indicates the location of the nested primer (LA8168)

Gene-specific primers (GSPs) for 3’RACE were designed to bind to Ex2a since it was common to all the isoforms identified (Fig. 5). In WT males, two different isoforms were found: the canonical isoform that spliced from Ex4 to Ex6 (Fig. 5A blue background), which was found with four different length UTRs, and an isoform which retained the intron between Ex4 and Ex5 and continued into Ex5 (Fig. 5A highlighted in brown). This latter isoform contained the target site for the *dsx*^*gRNA*^ cassette, leading to the identification of an isoform which transcribed through the intron between Ex4 and Ex5 and then into the transgene in homozygous males (Fig. 5C in pink). Nonetheless, due to a stop codon in the intron between Ex4 and Ex5, this isoform would code for 22 additional amino acids in the intronic sequence, with the Ex5/transgene sequence becoming part of the UTR. In homozygous males, the canonical isoform was not found, only the previously mentioned isoform which reads through the intron between Ex4 and Ex5 and into the transgene and two additional isoforms that splice into two different points of the Hr5/IE1 promoter (Fig. 5C highlighted in green). All the isoforms found in homozygous males would result in the disruption of the C-terminus of the male-specific canonical isoform of DsxM. These results suggest that the reduction in fertility observed in homozygous males might be due to this truncated protein. However, the relative abundance of these various isoforms is hard to assess since we could detect the canonical splicing when using primers in Ex4 and Ex6 in homozygous males by RT-PCR (Fig. S7).

Four different isoforms were identified in WT females (Fig. 5B): (i) an isoform which retained the intron between Ex3 and Ex4 (grey); (ii) an isoform that retained the intron between Ex4 and Ex5 which was also observed in WT males (brown); (iii) the canonical isoform which splices from Ex4 to Ex5 (yellow); (iv) an isoform that splices from Ex4 to what we termed Ex5b (dark yellow), which was also identified by RT-PCR (Fig. S7). Although an isoform that presented the entire sequence of Ex5 and then spliced into Ex6 was found in the RT-PCR (Fig. S7), we were not able to identify it by RACE. Further analysis of the sequences that spliced from Ex4 to Ex5 revealed that some sequences stopped at an A-rich repetitive region (Fig. 5B), suggesting that the absence of a sequence containing the entire Ex5 might have been due to a RACE PCR artefact where this A-rich region was recognized by the oligo-dT in the reverse transcription reaction. The first three isoforms seen in WT females were also identified in homozygous females. Similar to homozygous males, an isoform (highlighted in pink) transcribed through the *dsx*^*gRNA*^ cassette, and the other isoform spliced into the transgene instead of Ex5 (Fig. 5D green). Although isoforms that splice from Ex4 to Ex5b, which should not be affected by the presence of the transgene, were not observed in this experiment, they were identified in the previously described RT-PCR (Fig. S7), suggesting that the *Asdsx* female-specific splicing might not be fully disrupted by the transgene insertion. However, since both isoforms were fully or partially disrupted by the insertion, it is possible that the observed intersex phenotype was due to the disruption of the canonical female-specific intron-Ex5a boundary alone and not because of some additional disruption of the intron-Ex5b isoform due to the prevalence of isoforms which splice into the transgene insertion.

In summary, we have identified that both Ex2 and Ex5 differ from the annotated *Agdsx* transcript, in a similar manner, with one isoform which contains the entire exon and a second version which indicates an intron spliced out of this region. We also consistently found transcripts which read through the intron between Ex4 and Ex5, which if translated would result in a truncated protein, lacking the C-terminus in both males and females. Finally, all the isoforms identified in homozygous males and females would also result in a truncated protein lacking the C-terminus by either reading through or splicing into the transgene. These results might explain the observed phenotype.

## Discussion

In this study, we generated the first population suppression split drive in *An. stephensi.* The generated system consisted of a gRNA-expressing cassette targeting the female-specific exon of the *Asdsx* gene and a Cas9-expressing cassette under the control of the endogenous *zpg* promoter. Trans-heterozygous adult mosquitoes in the presence of *zpg*^*5’Cas9*^ were able to pass the *dsx*^*gRNA*^ cassette to > 98% of the progeny, indicating that high homing rates can be obtained in a split drive system and using the *Aszpg*-based expression. However, a reduction in fertility and fecundity was observed in trans-heterozygous females likely due to somatic expression and/or deposition of Cas9 in the embryo, resulting in conversion of the WT allele into a null, as was also observed in *An. gambiae dsx*-targeting drives [28]. A greater reproductive fitness cost was observed in females that received the Cas9 maternally, which differs from the paternal deposition observed in *An. gambiae* [10]. This additional fitness cost associated with somatic expression or deposition of the nuclease could weaken the ability of the drive to spread in a wild population. Therefore, reducing somatic expression and/or deposition of the nuclease is essential for gene drive efficiency. In recent years, efforts have been focused on restricting the spatiotemporal expression of the Cas9 nuclease [10, 12, 28, 38, 39], which has led to the identification and assessment of different germline promoters to express Cas9 such as *nanos* and *zpg*, improving this technology. Even though *nanos*-Cas9 and *zpg*-Cas9 *An. gambiae* transgenic mosquitoes have shown highly efficient drive performance [10, 28, 40], results shown here and by Terradas et al. [38] indicate that these promoters may cause parental deposition and/or ectopic expression of the Cas9 nuclease, especially when inherited from transgenic females. This deposition/ectopic expression of Cas9 results in conversion of some cells from heterozygous to homozygous and, in the case of *dsxF* as a target, result in a mosaic and at least partially intersex sterile female [41, 42]. Alternatively, *oskar* has been suggested as a potential Cas9 promoter in *Anopheles* mosquitoes since its expression was limited to the oocyte pole, where germ cells form, in females and to premeiotic regions of the testes in males [38].

Disruption of the splicing acceptor of the *dsx* female-specific exon in *An. gambiae* resulted in homozygous null females presenting an intersex phenotype, while males and heterozygous females remained fertile [10]. This research shows that disruption at the same site in *An. stephensi* resulted in a reduction of male and female fertility. The models of Deredec et al. [43] can be used to estimate the effects of such fitness costs on a gene drive system. Similar observations were reported by Xu et al. [42] where disruption of the female-specific isoform *Asdsx* resulted in homozygous males also presenting an intersex phenotype and being sterile. In both cases, the fitness appeared to be attributed to the transgene and not because of generated mutations since somatic cutting did not affect the fertility of heterozygous males. Results presented here indicate that the observed fitness reduction in homozygous males could be explained by the partial disruption of the splicing pattern and raise the question of whether the phenotype observed in homozygous females was uniquely due to the disruption of the intron-exon boundary or because of the additional potential interruption of the isoform which splices from Ex4 to Ex5b. These isoforms, if translated, should contain the N’ terminal DNA binding domain [44] but may interfere with dimerization domains and likely have ablated functions but more work is necessary to understand the many isoforms identified here. The presence of a female-specific isoform containing a shorter version of Ex5 (Ex5b) has been observed in *Aedes* mosquitoes, where the female-specific exon is divided into Ex5a and Ex5b [45], and recently in *An. stephensi* mosquitoes of the UCISS2018 strain [46]. Even though this isoform seems to be conserved within different *An. stephensi* strains, understanding whether it is a functional isoform or not might give a better understanding of sex determination in this anopheline species. Unlike in homozygotes, the reproductive fitness cost observed in heterozygous males and females cannot be attributed to the disruption of the splicing pattern if *dsx* is a fully haplosufficient gene in *An. stephensi* and the transgene represents an amorphic or hypomorphic mutation, as intended. Rather, these results are more consistent with the transgene acting as a dominant negative (antimorphic) mutant, which has been previously reported in *Drosophila suzukii* [41]*, Drosophila melanogaster* [47] and *An. gambiae* [48] where females expressing a drive were intersex and sterile. Although results shown by RACE suggest that changing the Hr5/IE1 component to an alternative promoter could improve the fitness of the *dsx*^*gRNA*^ mosquitoes, similar results observed in other insect species as well as those described here in *An. stephensi* using different construct designs suggest that disruption of *dsxF* by a knock-in might be more complex than previously anticipated. The enhancers found in this promoter may also be playing a role in the observed fitness effects of this transgene, potentially altering the expression level or pattern of the disrupted DSX protein. A more thorough investigation into the isoforms of *Asdsx* which are responsible for functional proteins should be undertaken to aid in rational construct design for gene drives targeting this gene.

## Conclusions

Our research yields insights for the development of population suppression gene drives targeting *dsx* in another anopheline species. It suggests that genes might have relevant biological differences in different mosquito species that might make the translation of previously developed and optimised genetic control tools to other vectors of interest difficult. Therefore, there is a need for a better understanding of the function of potential gene targets, such as *dsx*, in relevant mosquito vectors to develop control strategies suitable for different species. In addition, further investigation into more germline restricted promoters for Cas9 would minimise the effect of deposited/ectopically expressed Cas9 and should reduce fitness effects.

## Supplementary Information

Supplementary Material 1.

## Data Availability

The data supporting the conclusions of this article are included within this published article and its Supporting information files and the research data repository of the University of York: at https://doi.org/10.15124/77dd2bf5-06a6-4878-8f17-3423c1a98480. Raw data and analysis scripts are available at https://github.com/Philip-Leftwich/split-drive-dsx-Anopheles-stephensi
