# Supplementary material for: Challenges in developing a split drive targeting dsx for the genetic control of the invasive malaria vector Anopheles stephensi
Source: Parasit Vectors. 2025 Feb 7;18:46. doi: 10.1186/s13071-025-06688-0 (PMC11806748; doi:10.1186/s13071-025-06688-0)
Supplement: Supplementary file 1 — Supplementary Material 1. [file 13071_2025_6688_MOESM1_ESM.docx]

**Supplementary information**

**Methods**

Larva to adult survival

To determine the larva to adult survival of mosquitoes carrying the *dsx^gRNA^* allele, the offspring of *dsx^gRNA^* heterozygous females crossed to *dsx^gRNA^* heterozygous males was hatched in two different pools of approximately 200 larvae and screened for the presence of the ZsGreen fluorescent marker at L4 stage to determine the transgenic ratios. Positive larvae from one of the pools were collected and their genotype was determined by PCR amplification using the Phire tissue direct PCR master mix (Thermo Fisher Scientific) and the LA4341 and LA4347 primers. The remaining positive and negative larvae were reared separately under standard conditions. The number of females and males that reached pupae and adult stage were counted. Mosquitoes that reached adulthood were collected for gDNA extraction using the Phire tissue direct PCR master mix kit (Thermo Fisher Scientific). Their genotype and sex was determined by PCR amplification using the same kit. The LA4341 and LA4347 primers were used to determine the genotype and the LA7518 and LA7520 primers were used to determine the sex.

RT-PCR

RNA was extracted from homozygous, heterozygous and WT *dsx^gRNA^* adult males and females using the NucleoSpin RNA mini kit (Macherey-Nagel). The retrieved RNA was DNase treated with the TURBO DNA-free kit (Thermo Fisher Scientific) and used to synthesise cDNA with the LunaScript RT Supermix kit (NEB). No-RT controls were performed for each sample. cDNA and no-RT controls were PCR amplified using the LA7647 and the LA7649 primers and the Q5 Hot Start High-Fidelity 2x master mix (NEB) and analysed by agarose gel electrophoresis.

**Supplementary tables**

| Construct name | # injected G_0_s | # survivors (%) | # G_1_ screened | # positive G_1_s | # independent events |
| --- | --- | --- | --- | --- | --- |
| AGG2073 | 722 | 42 (5.82) | 7454 | 159 | 2 (out of 5 pools) |
| AGG2288 | 1747 | 141 (8.1) | 40127 | 1 | 1 (out of 7 pools) |

**Table S1. Summary of embryo injections.**

| Homozygotes | Heterozygotes | WT | Chi-squared value | p-value |
| --- | --- | --- | --- | --- |
| 30 | 68 | 38 | 0.4722 | 0.7897 |

**Table S2. No significant differences were observed in the genotypic ratio at L4 stage.** Statistical difference was calculated using the chi square test at a significance level of 0.05.

| Developmental stage | # Transgenic | # Non-transgenic | p-value |
| --- | --- | --- | --- |
| Larva | 164 | 58 | 0.9135 |
| Pupa | 149 | 47 | 0.9065 |
| Adult | 130 | 42 | 1 |

**Table S3. The progeny of *dsx^gRNA^* heterozygous females crossed to heterozygous *dsx^gRNA^* males did not present any significant differences in the transgenic ratio.** Statistical significance was measured through multiple Fisher exact tests at a 0.05 significance level in comparison to the 3:1 expected transgenic ratio. The genotype of the transgenic mosquitoes was characterised by PCR at adult stage. Out of the 130 transgenic mosquitoes that reached adulthood, 38 (29.23%) were homozygotes and 92 (70.77%) were heterozygotes, which was not significantly different from the expected 1:2 ratio (Fisher exact test p-value of 0.59 at 0.05 significance level).

| Genotype | # Females | # Males | p-value |
| --- | --- | --- | --- |
| WT | 20 | 22 | 1 |
| Heterozygotes | 44 | 48 | 0.8828 |
| Homozygotes | 15* | 23 | 0.4892 |

**Table S4. The progeny of *dsx^gRNA^* heterozygous females crossed to heterozygous *dsx^gRNA^* males did not present any significant differences in the transgenic ratio.** Statistical significance was calculated using multiple Fisher exact tests at a 0.05 significance level in comparison to the expected 1:1 sex ratio. *Females presenting an intersex phenotype.

| **Coefficient** | **Estimate** | **SE*^1^*** | **z** | **p-value** |
| --- | --- | --- | --- | --- |
| **Poisson model** | | | | |
| **(Intercept)** | 4.9 | 0.109 | 44.6 | **<0.001** |
| **line** |  |  |  |  |
| WT | — | — | — |  |
| Het | -0.22 | 0.126 | -1.72 | 0.085 |
| Hom | -0.57 | 0.163 | -3.53 | **<0.001** |
| **parent** |  |  |  |  |
| male | — | — | — |  |
| female | -0.12 | 0.126 | -0.937 | 0.35 |
| **Binomial model** | | | | |
| **(Intercept)** | -1.6 | 0.344 | -4.77 | **<0.001** |
| **line** |  |  |  |  |
| WT | — | — | — |  |
| Het | 1.0 | 0.360 | 2.87 | **0.004** |
| Hom | 2.7 | 0.490 | 5.59 | **<0.001** |
| **parent** |  |  |  |  |
| male | — | — | — |  |
| female | 0.62 | 0.358 | 1.74 | 0.082 |
| *This is a zero-inflated poisson mixed model* | | | | |
| *^1^* SE = Standard Error | | | | |

**Table S5. Zero-inflated Poisson error generalised linear mixed model of egg laying rates from *dsx^gRNA^* mosquitoes crossed to SDA-500 mosquitoes.** Mixed effects models included replicate as a nested random factor within each cross.

| **Coefficient** | **Estimate** | **SE*^1^*** | **z** | **p-value** |
| --- | --- | --- | --- | --- |
| **(Intercept)** | 0.93 | 0.680 | 1.37 | 0.17 |
| **line** |  |  |  |  |
| WT | — | — | — |  |
| Het | 0.39 | 0.963 | 0.401 | 0.69 |
| Hom | -0.48 | 0.927 | -0.515 | 0.61 |
| **parent** |  |  |  |  |
| male | — | — | — |  |
| female | 0.21 | 0.962 | 0.220 | 0.83 |
| **line * parent** |  |  |  |  |
| Het * female | -4.0 | 1.39 | -2.91 | **0.004** |
| Hom * female |  |  |  |  |
| *This is a binomial mixed model* | | | | |
| *^1^* SE = Standard Error | | | | |

**Table S6. Binomial generalised linear mixed model of egg hatching rates from *dsx^gRNA^* mosquitoes crossed to SDA 500 mosquitoes.** Mixed effects models included replicate as a nested random factor within each cross.

| Cas9 parent | #F_1_ *dsx^gRNA^* positives | # F_1_ *dsx^gRNA^;zpg^5’Cas9^* positives | # F_1_ *zpg^5’Cas9^* positives | # WT F_1_ |
| --- | --- | --- | --- | --- |
| Female | 246 | 190 | 214 | 230 |
| Male | 191 | 227 | 239 | 228 |

**Table S7.** Inheritance rate of F_1_ progeny from the *dsx^gRNA^* x *zpg^5’Cas9^* F_0_ crosses.

| Cas9 parent | Engorgement | Survival to blood meal | Egg laying | Hatching |
| --- | --- | --- | --- | --- |
| Male |  |  |  |  |
| Male trans-het | 43/48 | 42/43 | 39/41 | 38/39 |
| Female trans-het | 37/46 | 36/37 | 13/36 | 8/13 |
| Female |  |  |  |  |
| Male trans-het | 49/49 | 39/49 | 33/39 | 30/33 |
| Female trans-het | 12/44 | 10/12 | 2/12 | 0/2 |

**Table S8. Itemization of all the stages between blood-feeding and hatching rate that contribute to a reproductive phenotype of the trans-heterozygous F_1_ females used in the homing assay.** In each stage, females unable to contribute to the next one were excluded. Trans-het: trans-heterozygote for the *dsx^gRNA^* and *zpg^5’Cas9^* alleles.

| **Coefficient** | **Estimate** | **SE*^1^*** | **z** | **p-value** |
| --- | --- | --- | --- | --- |
| **Poisson model** | | | | |
| **(Intercept)** | 4.5 | 0.475 | 9.49 | **<0.001** |
| **Cas9_parent** |  |  |  |  |
| Cas9_parentMale | 0.15 | 0.489 | 0.302 | 0.76 |
| **Cas9_grandparent** |  |  |  |  |
| Cas9_grandparentMale | -0.70 | 0.507 | -1.38 | 0.17 |
| **Cas9_parent * Cas9_grandparent** |  |  |  |  |
| Cas9_parentMale * Cas9_grandparentMale | 0.56 | 0.532 | 1.05 | 0.30 |
| **Binomial Model** | | | | |
| **(Intercept)** | 2.9 | 0.726 | 4.02 | **<0.001** |
| **Cas9_parent** |  |  |  |  |
| Cas9_parentMale | -4.6 | 0.851 | -5.43 | **<0.001** |
| **Cas9_grandparent** |  |  |  |  |
| Cas9_grandparentMale | -2.2 | 0.792 | -2.81 | **0.005** |
| **Cas9_parent * Cas9_grandparent** |  |  |  |  |
| Cas9_parentMale * Cas9_grandparentMale | 2.1 | 1.01 | 2.04 | **0.041** |
| *This is a zero-inflated poisson mixed model* | | | | |
| *^1^* SE = Standard Error | | | | |

**Table S9. Zero-inflated Poisson error generalised linear mixed model of egg laying rates from *dsx^gRNA^; zpg^5’Cas9^*  trans-heterozygous mosquitoes crossed to SDA 500 mosquitoes.** Mixed effects models included replicate as a nested random factor within each cross.

| Coefficient | Estimate | SE*^1^* | z | **p-value** |
| --- | --- | --- | --- | --- |
| **(Intercept)** | -0.22 | 0.745 | -0.297 | 0.77 |
| **Cas9_parent** |  |  |  |  |
| Cas9_parentMale | 1.7 | 0.677 | 2.47 | **0.014** |
| **Cas9_grandparent** |  |  |  |  |
| Cas9_grandparentMale | 0.36 | 0.425 | 0.846 | 0.40 |
| **Cas9_parent * Cas9_grandparent** |  |  |  |  |
| Cas9_parentMale * Cas9_grandparentMale |  |  |  |  |
| *^1^* SE = Standard Error | | | | |

**Table S10. Binomial generalised linear mixed model of egg hatching rates from *dsx^gRNA^; zpg^5’Cas9^* trans-heterozygous mosquitoes crossed to SDA 500 mosquitoes.** Mixed effects models included replicate as a nested random factor within each cross.

| **Coefficient** | **Estimate** | **SE*^1^*** | **z** | **p-value** |
| --- | --- | --- | --- | --- |
| **(Intercept)** | 6.5 | 1.22 | 5.28 | **<0.001** |
| **Cross** |  |  |  |  |
| `F1 cross`SDA-500x(2073:2288) | -1.2 | 1.25 | -0.951 | 0.34 |
| `F1 cross`SDA-500x(2288:2073) | -1.9 | 1.25 | -1.56 | 0.12 |
| *This is a binomial mixed model* | | | | |
| *^1^* SE = Standard Error | | | | |

**Table S11. Binomial generalised linear mixed model of inheritance rates from *dsx^gRNA^; zpg^5’Cas9^* trans-heterozygous mosquitoes crossed to SDA 500 mosquitoes.** Mixed effects models included replicate as a nested random factor within each cross.


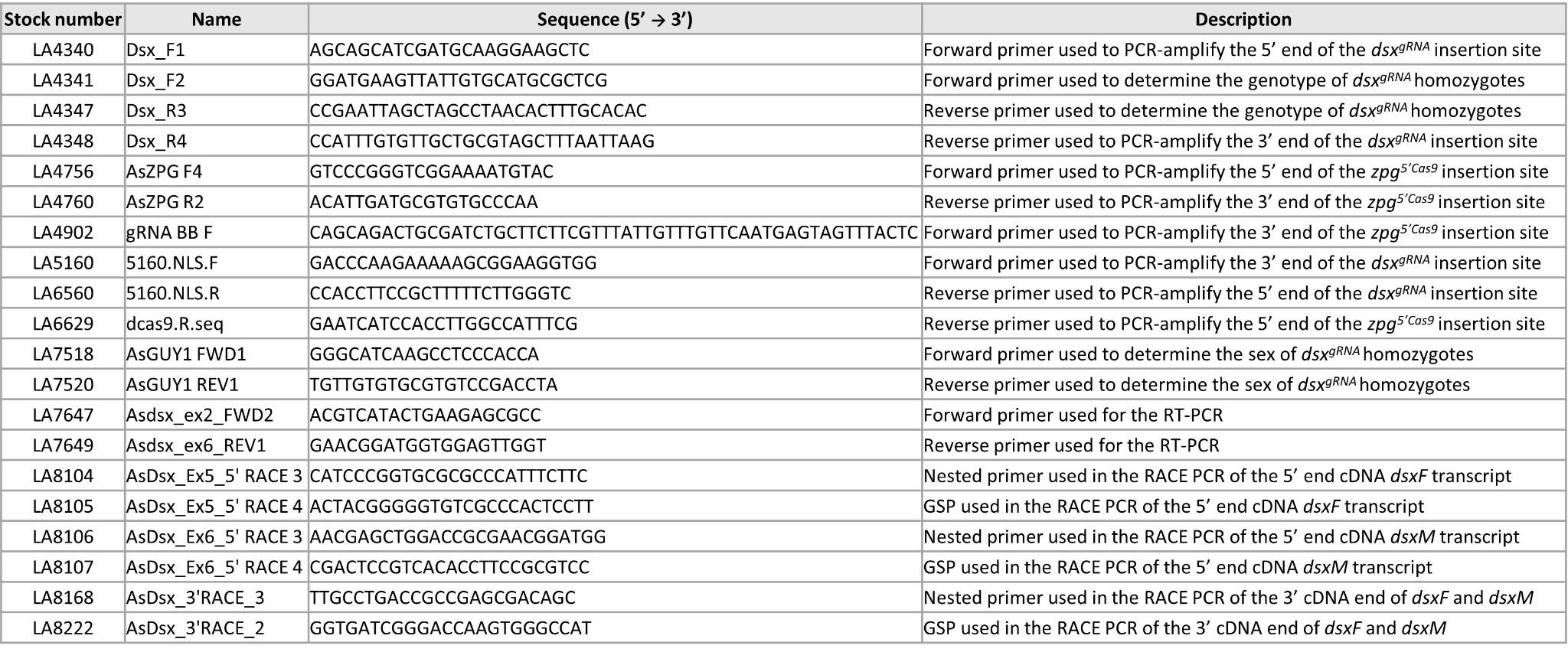


**Table S12. List of the primers used in this study.**

**Supplementary figures**


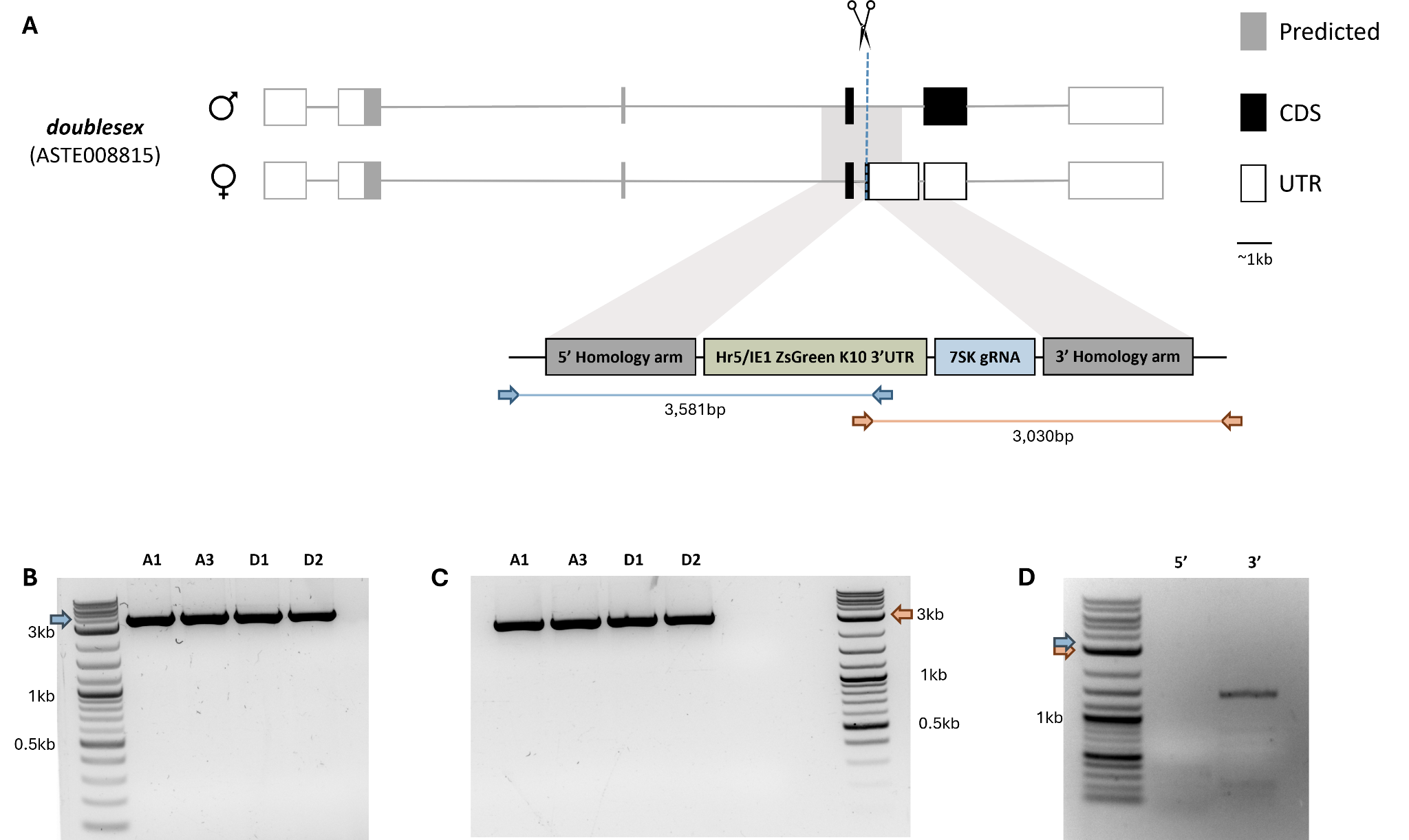


**Fig S1. Insertion site PCR of four isolines.** (A) Schematic representation of the male- and female-specific *dsx* transcripts in *An. stephensi* including the gRNA sequence, and the HDR knock-in construct with its corresponding insertion site locus. The black region of the *Asdsx* transcript was retrieved from Vectorbase (ASTE008815), whereas the remaining sequence (grey) was predicted from *An. gambiae* (AGAP004050). The expressed gRNA was designed to target the intron 4-exon 5 boundary. The cut site of the gRNA is indicated by scissors and the protospacer-adjacent motif (PAM) is highlighted in blue. Exons are drawn approximately to scale, whereas introns are not. Blue arrows represent the primers used for the 5’ PCR (LA4340 and LA6560) and the expected amplicon size, whereas the orange arrows represent the primers used for the 3’ PCR (LA5160 and LA4348). (B) Amplicons observed after PCR amplification of the 5’ amplicon. Each lane represents a different isoline used as a template. The blue arrow indicates the expected location of the amplicon. (C) Amplicons observed after PCR amplification of the 3’ amplicon. Each lane represents a different isoline used as a template. The orange arrow indicates the expected location of the amplicon. (D) PCR amplification of SDA-500 genomic DNA used as a negative control. The blue and the orange arrows point at the expected location of 5’ and 3’ the amplicons, respectively. The 1 kb plus DNA ladder (NEB) was used as a reference to determine amplicon size.


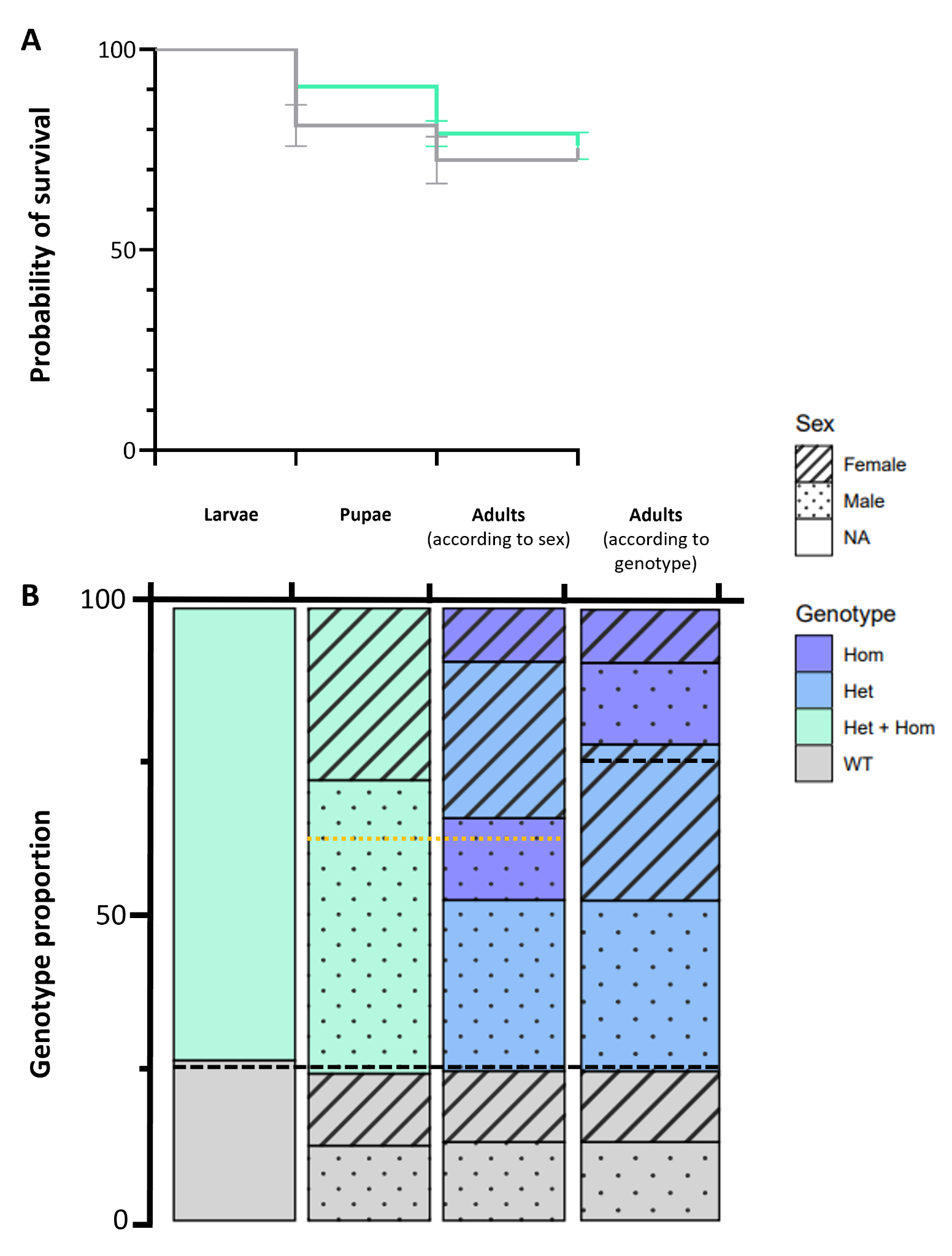


**Fig S2. Larvae to adult survival.** (A) Survival curve of transgenic mosquitoes (green) and non-transgenic mosquitoes (grey) from larvae to adult stage. A Mantel-Cox test was performed to assess if the observed difference was significant. A chi-square value of 0.47 and a p-value of 0.49 were obtained for a 0.05 significance level. No significant difference was observed for any of the performed tests. (B) Percentage of each screened phenotype and/or genotype at every life stage. WT or non-transgenic mosquitoes are represented in grey, transgenic mosquitoes (homozygotes and heterozygotes combined during developmental stages when they could not be distinguished) are shown in green, heterozygotes are represented in light blue, and homozygotes are shown in dark blue. The black dashed line indicates the expected distribution for WT mosquitoes and the three genotypes at adult stage. The orange dotted line indicates the expected sex ratio at pupae and adult stage for transgenic mosquitoes. Raw data used for this figure and statistical analysis can be found in Table S3.


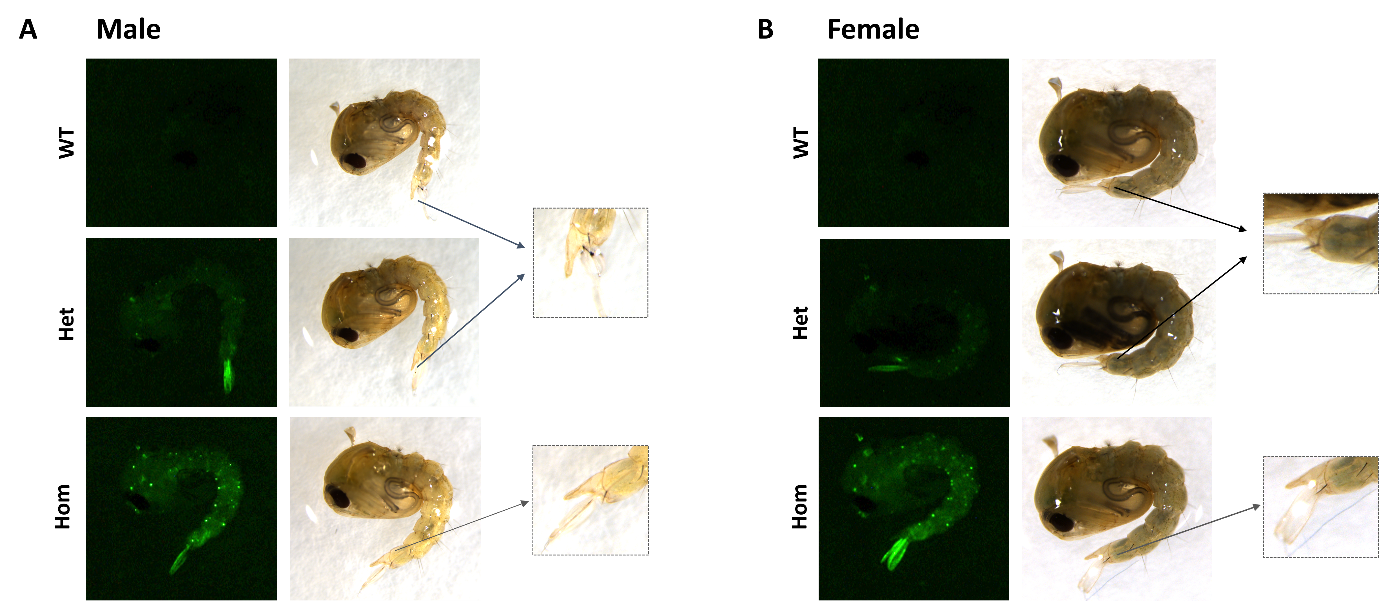


**Fig S3. Morphological appearance of dsx^gRNA^ homozygotes, heterozygotes and WT at pupae stage.** Imaging of male and female homozygotes, heterozygotes, and WT at pupae stage. From left to right for each identified sex we can observe the fluorescent pattern of the different genotypes, colour imaging of the whole pupae, and magnification of the pupae genitalia. No morphological differences were observed in males expressing the *dsx^gRNA^* cassette in comparison to WT males. Regarding females, no morphological differences were observed in heterozygous pupae in comparison to WT. However, all the screened homozygous pupae exhibited male-like genital lobes. The camera parameters used for fluorescent images were 889.31ms of exposure, gain 2, and 0.6x magnification. For colour images we used 70.34ms of exposure, gain 1, and a magnification of 1.6x instead.


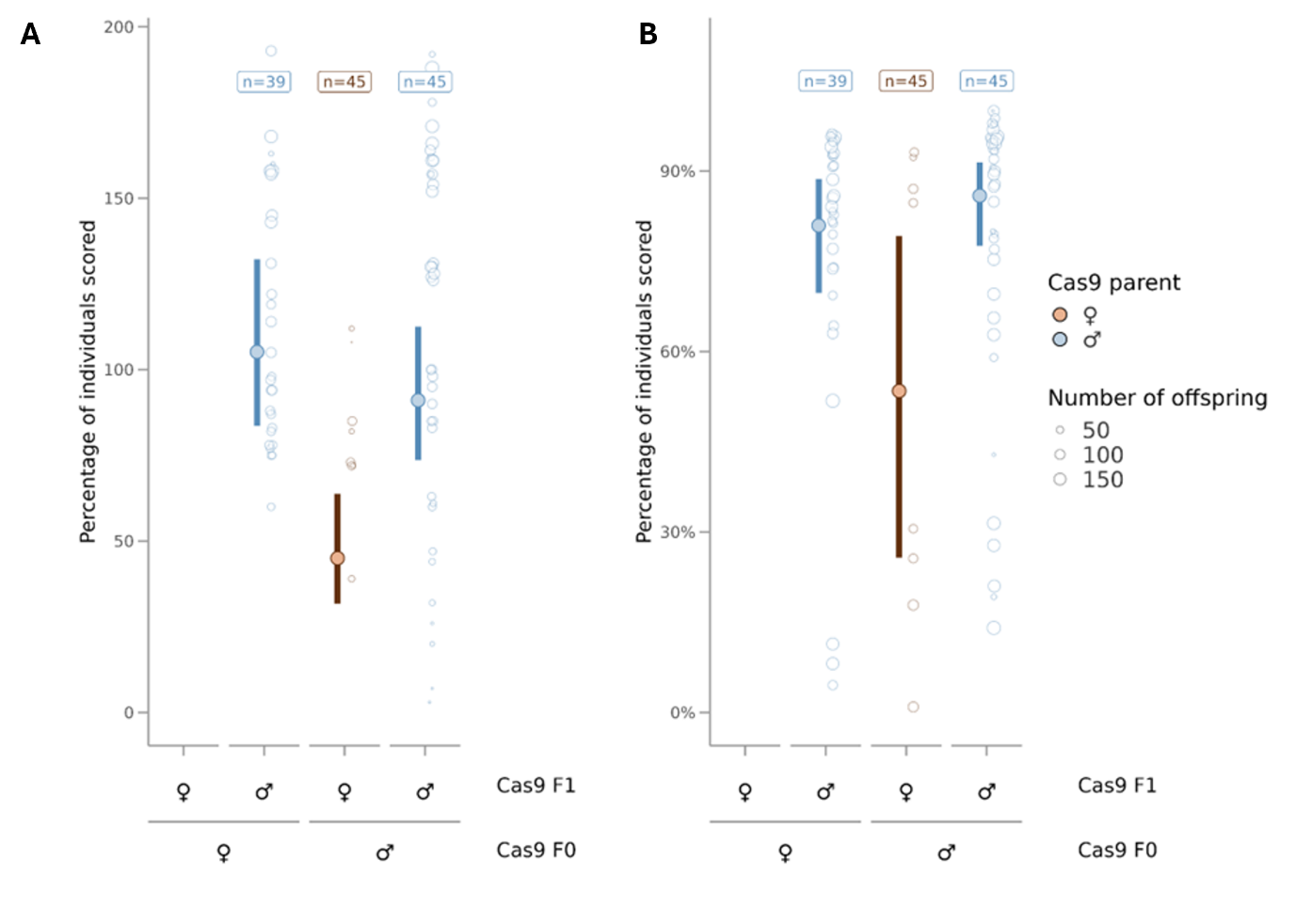


**Fig S4. Analysis of the reproductive fitness of mosquitoes carrying the Cas9 and dsx^gRNA^ allele.** A) Number of eggs that were laid per female. Individual faded points represent the number of offspring from a single parent. Large symbols and error bars (vertical lines) represent estimated mean and 95% confidence intervals calculated by a zero-inflated generalized linear mixed model, with a poisson (‘log’ link) error distribution (B) Hatching rate of the eggs laid by each female (number of hatched larvae was divided by the number of eggs and multiplied by 100). Individual faded points represent the number of offspring from a single parent and the size of the point is proportional to the number of offspring from the parent. Large symbols and error bars (vertical lines) represent estimated mean and 95% confidence intervals calculated by a generalized linear mixed model, with a binomial (‘logit’ link) error distribution.


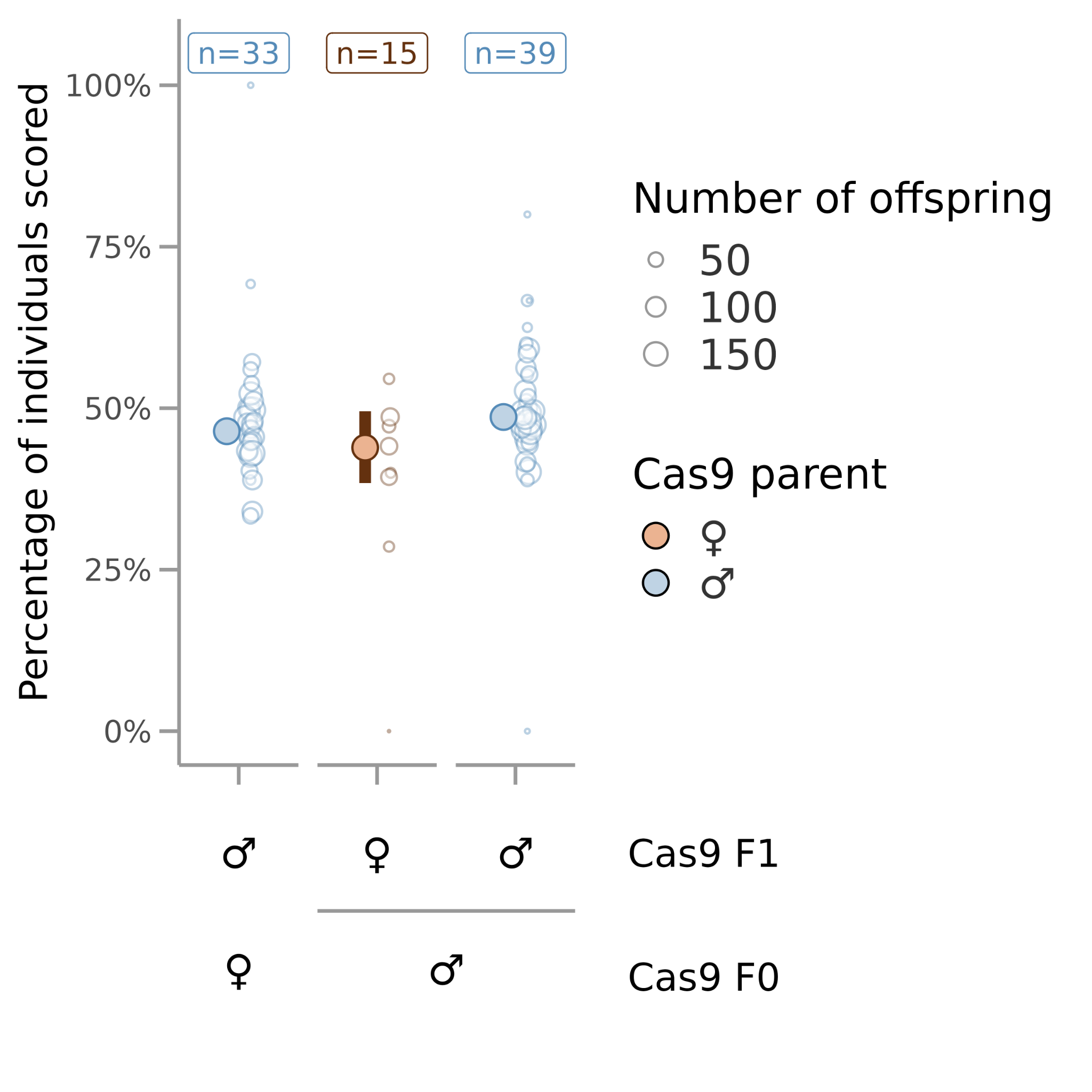


**Fig S5. Inheritance rate of *zpg^5’ Cas9^*.** Percentage of F2 larvae that inherited the zpg^5’ Cas9^ element. Open circles represent the inheritance rates from a single female and their size is proportionate to the number of offspring. The progeny of trans-heterozygous females is represented in orange and the offspring of trans-heterozygous males in blue. Filled points and error bars represent the estimated mean and the 95% CI calculated by a generalized linear mixed model, with a binomial (‘logit’ link) error distribution.

**
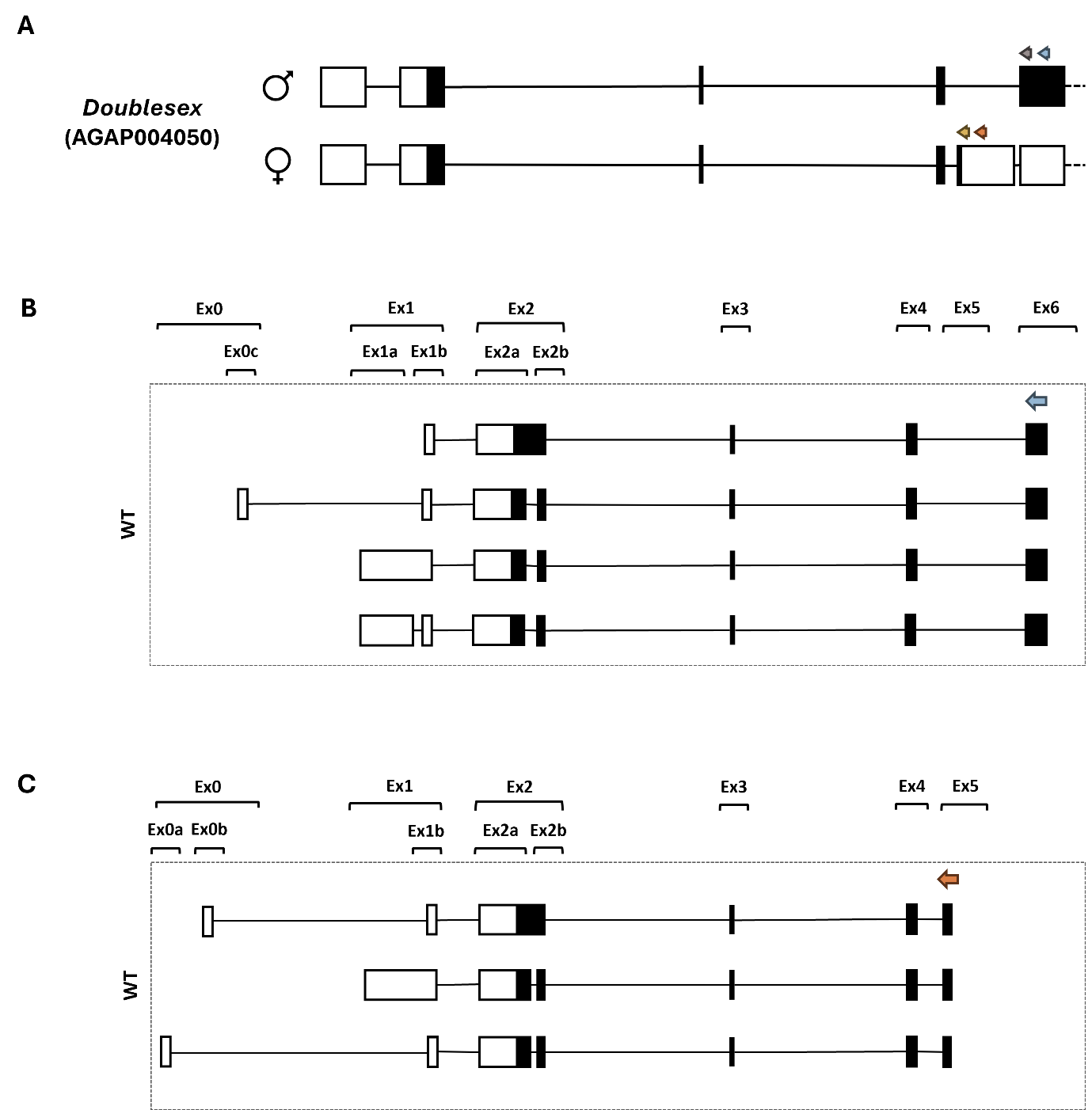
**

**Fig S6. Isoforms identified for the 5’ end of the *dsxM* and *dsxF* cDNA transcripts.** (A) Location of the GSPs on the *Asdsx* gene (ASTE008815) used to PCR amplify the 5’ end of the cDNA transcript. The GSPs LA8104 and LA8105, represented by yellow and orange arrows respectively, were designed to align to Ex5 and were used on the female DNA templates. The green and blue arrows correspondingly indicate the location of the LA8106 and LA8107 primers in Ex6. These primers were used to PCR-amplify the male samples. (B) Isoforms obtained from the 5’ end of the *dsxM* cDNA transcript. The blue arrow represents the location of the GSP primer (LA8107). (C) Isoforms obtained from the 5’ end of the *dsxF* cDNA transcript. Exon numbers are indicated at the top. The orange arrow indicates the location of the GSP primer (LA8105). Only sequences that included the UPM sequence at the 5’ end were included in the analysis.

**
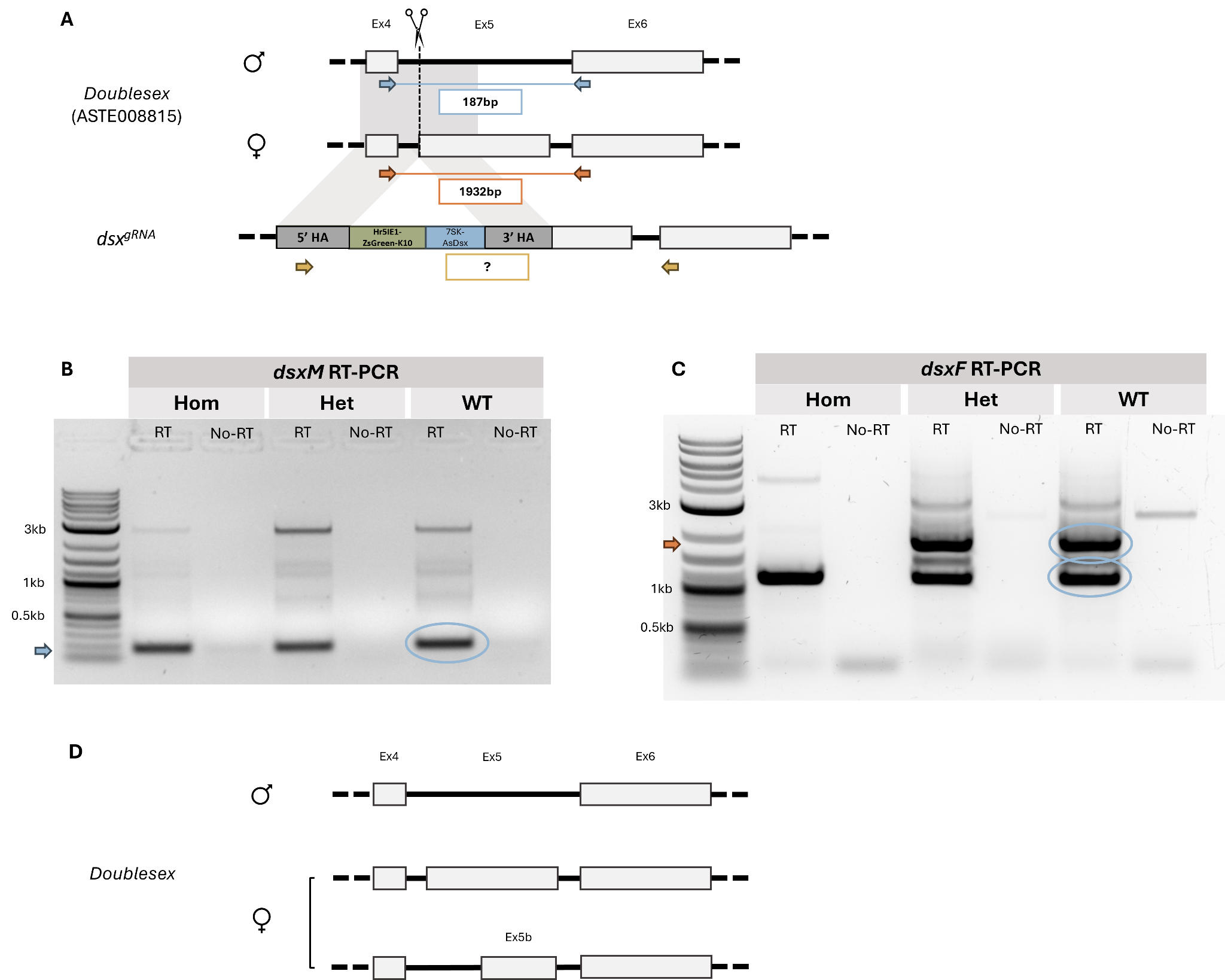
**

**Fig S7. RT-PCR of the *dsx^gRNA^* insertion in WT, heterozygous and homozygous males and females.** (A) Schematic representation of the male- and female-specific *dsx* predicted isoforms (ASTE008815) with the designed primers and the expected amplicon sizes. All the arrows in the figure characterise the same pair of primers and the different colours represent different amplicon sizes. No amplicon was expected for the *dsx^gRNA^* knock-in (yellow) since the transcription was predicted to stop before reaching the reverse primer located in exon 6. (B) Gel electrophoresis of the RT-PCR of the male-specific isoform. The blue arrow indicates the location of the expected amplicon using the 1kb plus ladder (NEB) as a reference. The blue circle highlights the amplicon that was sequenced. No differences were observed between homozygous, heterozygous and WT males. (C) Analysis of the RT-PCR of the female-specific isoform using an agarose gel electrophoresis. The orange arrow indicates expected amplicon size. The 1kb plus ladder (NEB) was also used as a reference. WT and heterozygous females showed three additional amplicons which were not observed in homozygous females. The blue circles highlight the two amplicons that were sent for sequencing. (D) Schematic representation of the alignment performed with the sequences obtained from the RT-PCR and the ASTE008815 sequence. The alignment showed that splicing in males occurred from Ex4 to Ex6 as expected. In WT females, two isoforms were detected. While the larger amplicon aligned to the predicted female-specific Ex5, the smaller amplicon showed splicing from Ex4 to a second splice acceptor in Ex5, which has been named Ex5b. This isoform was also observed in homozygous females, suggesting that the generated knock-in was not able to completely disrupt the DsxF isoform. Hom: homozygotes; Het: heterozygotes; WT: wild-type; RT: presence of reverse-transcriptase; No-RT: absence of reverse-transcriptase. The No-RT columns were used to detect the presence of non-degraded DNA in each sample.
